# Advances in the Development of Biomarkers for Poststroke Epilepsy

**DOI:** 10.1155/2021/5567046

**Published:** 2021-04-17

**Authors:** Mengke Liang, Liren Zhang, Zhi Geng

**Affiliations:** ^1^Department of Neurology, Shanghai Jiao Tong University Affiliated Sixth People's Hospital South Campus, Shanghai 201499, China; ^2^Department of Neurology, Shanghai Jiao Tong University Affiliated Sixth People's Hospital, Shanghai 200233, China

## Abstract

Stroke is the main cause of acquired epilepsy in elderly people. Poststroke epilepsy (PSE) not only affects functional recovery after stroke but also brings considerable social consequences. While some factors such as cortical involvement, hemorrhagic transformation, and stroke severity are associated with increased seizure risk, so far that remains controversial. In recent years, there are an increasing number of studies on potential biomarkers of PSE as tools for diagnosing and predicting epileptic seizures. Biomarkers such as interleukin-6 (IL-6), tumor necrosis factor-*α* (TNF-*α*), glutamate, and S100 calcium-binding protein B (S100B) in blood are associated with the occurrence of PSE. This review is aimed at summarizing the progress on potential biomarkers of PSE.

## 1. Introduction

The main cause of seizures in adults beyond the age of 60 is cerebrovascular disease [1], and the leading are hemorrhagic and ischemic strokes. Poststroke epilepsy (PSE) is one of the complications that lead to poorer quality of life, higher mortality, and greater health expenditures.

Seizures are defined as a transient, abnormally excessive synchronous neuronal activity in the brain. Poststroke seizures occur within 1 week of stroke onset (as early-onset seizures) or 1 week after a stroke (as late-onset seizures) [2]. Studies reporting on the rate of early postischemic stroke seizures range from 2% to 33%, while that of late seizures spans from 3 to 67% [3]. Seizure activity is present in up to 8–13% of patients following intracerebral hemorrhage [4]. This variability in the reported incidences is likely due to different study designs and length of follow-up. Seizures can be used as a predictor of poor functional outcome or longer hospital stays in patients with stroke [5]. The classical factors such as cortical involvement, hemorrhagic conversion of ischemic stroke, and stroke severity carry a higher risk of seizures [5–7].

However, the mechanism of poststroke epilepsy is not well understood. The majority of available studies agree cellular ionic imbalance and excitotoxic neurotransmitters are the main cause of the early seizure, while the late seizure is associated with glial scar formation resulting in increased nerve excitability [8]. The disruption of the blood-brain barrier and brain tissue ischemia/hypoxia injury all can lead to ion pump dysfunction. The elevation of the intracellular calcium ion, sodium, and extracellular potassium ion concentration will cause neural hyperexcitability and reduce seizure threshold [1, 9, 10]. Ischemic injury caused astrocyte and microglial activation, which will increase the production of inflammation mediators including tumor necrosis factor-*α*, interleukin-1*β*, and interleukin-6. Abnormal immune responses aggravate the damage to the BBB integrity that may promote epileptogenesis [11, 12]. Disruption of the neurovascular unit integrity also induced albumin extravasation, which results in significant accumulation of extracellular glutamate by activating the TGF+ signaling pathways [13]. Glutamate is the primary excitatory neurotransmitter in the central nervous system. The neurotransmitter glutamate is neurotoxic inducing or contributing to epileptogenesis when it is accumulated in a massive amount in the extracellular fluid [14, 15].

Glial scar formation was closely associated with late seizures. Increased expression of gliosis in the ischemic area is accompanied by abnormal nerve conduction and heightened neural excitability [8]. Compared with ischemic stroke, those with hemorrhagic stroke had higher seizure frequency. This well-known phenomenon may account for the direct stimulation of blood components to the superficial cortex [16]. Animal models have emphasized the involvement of hemosiderin depositions in late seizure susceptibility and epileptogenesis. Besides, as the study of poststroke epilepsy is deepened, some epileptogenic genes are associated with seizure susceptibility and are possibly involved in epileptogenesis [17].

Diagnosis of poststroke epilepsy is often challenging because of the diversity of clinical manifestations and low recognition. Electroencephalography (EEG), not widely used at present in hospitals, is unable to provide definitive information to predict an impending seizure or to guide inpatient AED management [18]. In recent years, more researches have confirmed that the blood biomarkers are associated with the occurrence of PSE and even provide more accurate and reliable information for the prognostic of PSE. This review is aimed at summarizing the existing studies on potential biomarkers for PSE diagnosis and prognosis.

## 2. Inflammatory Biomarkers

Inflammatory mediators increasing amount of clinical studies suggest that it has a tight connection with the initiation and progression of poststroke epilepsy. Repeated seizures can trigger inflammatory response, which in turn stimulates the production of inflammatory factors. At the same time, a variety of inflammatory markers of the nervous system can increase neuronal excitability and induce seizures [19, 20].

### 2.1. Interleukin

Interleukin is a common cytokine that participates in inflammation and has a pivotal role in the systemic immunomodulatory. Although its content is at a low level in the human body, the expression of cytokines will increase rapidly when brain tissue suffers from ischemic injury and further result in an inflammatory reaction. Recent studies suggest that inflammatory mediators, such as interleukin- (IL-) 1*β*, IL-6, and IL-10, play a significant role in the underlying pathophysiology of epilepsy, contributing to the onset and perpetuation of seizures [21, 22]. Interleukin-6 (IL-6), regarded as the upstream mediator of the inflammatory reaction [23], is rapidly upregulated after acute CNS injury. The determination of the IL-6 level is a good predictor of poststroke epilepsy. Previous studies have found that patients with seizures resulting from multiple etiologies are accompanied by elevated levels of IL-6 [22, 24]. Kegler et al. found that Ala16Val MnSOD (alanine to valine at the 16th amino acid manganese superoxide dismutase) polymorphism has an important role on neuroinflammation maintenance and its consequences, especially the VV genotype in epilepsy patients, increased levels of inflammatory cytokines such as IL-1*β* and IL-6 [22]. This finding has also been confirmed in animal models [25]. Mice are highly vulnerable to seizures by intracranial injection of IL-6 into the neocortex [26, 27]. A cohort study of patients with acute ischemic stroke also found that the serum IL-6 levels in patients with poststroke epilepsy were significantly higher than those in patients without seizures, which could be regarded as an independent predictor of seizures in stroke patients. Jia et al. studied 209 patients with poststroke epilepsy who were diagnosed within 1 week of admission and found that serum IL-6 can be used as a biomarker of recurrent epilepsy after stroke. The results of the ROC curve showed that the possibility of predicting epilepsy recurrence by IL-6 was 70% [28]. Besides, IL-6 levels were also associated with the development of poststroke cognitive impairment ,patients with higher levels of IL-6 had lower MMSE scores three months after stroke [29].

Interleukin-1*β* is also a multifunctional cytokine produced by brain glial cells related to seizures. Multiple studies have found that various biological samples such as serum and cerebrospinal fluid of patients with epilepsy are accompanied by the increase of IL-1*β* [30–32]. The level of interleukin-1*β* in epileptogenic regions of the poststroke epileptic animal model was also significantly higher than that of normal tissue. The injection of exogenous IL-1*β* antibodies into the brain will have a prominent anticonvulsant effect on epileptic mice. Zhang et al. found that IL-1 was overexpressed in patients with recurrent poststroke epilepsy compared to the patients without recurrence. Receiver operating characteristic (ROC) curve analysis showed that the area under curves was 0.808 when the best cut-off value of IL-1*β* was 5.42, which could be used as a biomarker of recurrent epilepsy after stroke [33]. In addition, IL-1*β* is also the intermediate medium for IL-10 to participate in the epileptic response. With a strong anti-inflammation effect, IL-10 is known to suppress seizures in epilepsy patients by reducing the expression of interleukin-1 [34]. There is a significant negative correlation between plasma IL-10 levels and the prolongation of seizure time, while the continuous decrease of IL-10 has a significant positive correlation with hippocampal sclerosis in patients with epilepsy [35]. The significance of IL-10 in epilepsy remains controversial because some studies have reported that the level of IL-10 in patients with epilepsy is increased instead of decreased. A recent meta-analysis has noted that IL-8, IL-17, and IL-22, several inflammatory mediators that can lead to blood-brain barrier disruption, are also crucial factors involved in seizures by evaluating the correlation between epilepsy and different subtypes of interleukin [32]. Different experiments have demonstrated that in patients with epilepsy, serum IL-17 levels were 5-15 times higher than in the control groups [36], but there was no research on the correlation between poststroke epilepsy and the above three subtypes, which need to be further studied.

### 2.2. Tumor Necrosis Factor

Tumor necrosis factor (TNF) has multiple subtypes, among which TNF-*α* is the most highly correlated to epilepsy. Extra-CNS TNF-*α* is inaccessible to the intracranial brain tissues. However, it will not only stimulate glial cells to excessively secrete tumor necrosis factor TNF-*α* but also promote peripheral inflammatory cytokines transported from the blood into the brain when the integrity of the blood-brain barrier is impaired. The high level of tumor necrosis factor associated with the inflammatory response after stroke is an important cause of poststroke epilepsy [37]. Studies have noted increased TNF-*α* serum levels in individuals with epilepsy compared with normal subjects. The content of TNF-*α* in brain tissue of epilepsy rats similarly shows a significant upward trend [38]. Low-dose TNF-*α* (5.0 *μ*g/kg) significantly increased the frequency of epilepsy and prolonged the duration of neuronal firing [39, 40]. The content of TNF-*α* was positively correlated with the severity of epilepsy, which was higher in the severe epilepsy group than in the mild group, while the nonepileptic group is at a minimum level [41]. Tumor necrosis factor is mainly involved in the pathological process of seizures through two receptors, which will induce seizures by binding receptor 1, while the activation of receptor 2 will exert anticonvulsant effects. Abraira noted that low levels of tumor necrosis factor receptor 1 are an independent predictor of early poststroke seizures. The optimal cut-off value in the ROC curve is 0.013 with the strongest prognostic value, which can be interpreted as evidence of the increase of TNF-R1 binding after stroke. The study also pointed out that the combination of TNF-R1 with existing clinical predictors will significantly improve the early identification of poststroke epilepsy [42]. Despite no relevant research that has reported that TNF-R2 could be used to predict epilepsy, receptor 1 inhibition and receptor 2 activations may be an effective means for preventing poststroke epilepsy.

## 3. Neurotransmitter Biomarkers

The imbalance of neurotransmitters is a crucial cause of seizures. Glutamate is the primary excitatory neurotransmitter in the CNS, while GABA is a main inhibitory transmitter that counterbalances the excitatory action of glutamate. On the one hand, glutamic acid decarboxylase can convert glutamic acid to *γ*-aminobutyric acid, while *γ*-aminobutyric acid can be converted to *α*-keto succinate by a *γ*-aminobutyric acid transaminase. It will lead to the occurrence of poststroke epilepsy when stroke leads to the imbalance of the above circulatory metabolic pathways. High levels of glutamate in the acute phase of stroke are a biomarker for poststroke seizures [15, 43]. Xie et al. found that plasma glutamate levels in patients with poststroke epilepsy are significantly higher than those without seizures [14]. Animal experiments have similarly confirmed that the glutamate level in the central nervous system increased significantly when brain tissue suffering from ischemic damage especially the ischemic core region is most evident. Studies on patients with tumor-associated epilepsy also confirmed that glutamate in the epileptogenic hippocampus was significantly higher than that in other populations. Recent studies have found that the content of glutamate was significantly higher during the interictal period than during epileptic seizures in epilepsy patients who undergo surgical treatments. Thus, they suggest that increased levels of glutamate can result in the occurrence of spontaneous seizures. Stroke causes excessive glutamate released from the presynaptic membrane into the synaptic, which produces excitotoxicity leading to the recurrence of abnormal neuronal discharge [44]. Therefore, glutamate could be an important target to prevent or treat epilepsy. On the other hand, the expression of GABA and its receptors is downregulated after stroke, which reduces the seizure threshold and leads to the occurrence of epilepsy [14]. Patients who received oral GABA within 14 days after a stroke had higher GABA and lower glutamate levels than those without any intervention when remeasured one month after drug withdrawal. GABA administration can effectively inhibit the occurrence of early poststroke epilepsy. The decrease in GABA production and GABA receptor expression equally provide reliable information for the prediction of poststroke epilepsy.

## 4. Genetic Biomarkers

Since the first seizure-related gene was found in animal experiments, more than 500 genes and loci have been confirmed to contribute to the seizures. To the best of our knowledge, approximately 30% of epilepsy syndromes have been shown to be related to genetic factors, several of which have been identified as causally related to poststroke epilepsy [17].

### 4.1. ALDH2 rs671

ALDH2 rs671 polymorphism, a reliable index that can be used to predict poststroke epilepsy, refers to the mutations in exon 12, which result in the substitution of glutamic acid by lysine. Therefore, ALDH2 can be divided into three different genotypes: homozygous mutations (AA genotype), heterozygous mutations (GA genotype), and wild-type mutations (GG genotype) [45]. The mutations of the A gene could lead to a decrease of 90% in the enzyme activity of ALDH. A case-control study found that rs671A allelic or genotypic frequencies in the PSE group were significantly higher than those in the IS group, and the difference between the two groups was striking. ALDH2 is a key enzyme in 4HN metabolism, inducing seizures by suppressing ALDH2 activity that may lead to decreased proteolysis of 4HN. The mutated genes induce seizures by suppressing ALDH2 activity that may lead to decreased proteolysis of 4HN. 4-HNE serves as a specific biomarker for oxidative stress. The continuous increase of 4-HNE promotes inflammatory reactions, which enhance free radical production, increase excitability of neurons, and induce epilepsy [46]. Increased 4-HNE levels were found in mice with middle cerebral artery occlusive cerebral infarction [47]. Zhang confirmed that patients with poststroke epilepsy showed a significantly higher 4-HNE level in the postacute phase compared with the levels in patients with stroke who did not develop PSE and in control patients. There is a positive correlation between 4-HNE and PSE, which suggests that the content of serum 4-HNE provides a convinced index for evaluating the risk of epileptic seizures after stroke [48]. ALDH2 is one of the best-characterized target proteins of daidzein. The combination of the two acts as an antiepileptic effect by multiple pathways [49].

### 4.2. TRPM6 Polymorphism

There is a close relationship between cell electrophysiological instabilities and epilepsy, and varying the concentration of the ion inside and outside of the cell has been identified to be the link between them. Decreased serum magnesium was demonstrated to reduce seizure threshold or increase seizure susceptibility, leading to an increased vulnerability to epilepsy [50]. Magnesium sulfate (MgSO_4_) is the most commonly used medication for the control of seizure in preeclampsia for which the patient is at risk. N-Methyl-D-aspartate-induced convulsions in experimental animals can be effectively controlled by oral magnesium supplementation [51].

Transient receptor potential cation channel subfamily M member 6 (TRPM6), belonging to nonselective cation channel families, is the key regulator responsible for the transportation of the body's Mg2+ balance [52]. Mutation of the gene encoding TRPM6 can decrease blood magnesium levels due to the imbalanced ion transport in intestinal and kidney tubule epithelia. Some studies have reported that a homozygous variant genotype of the polymorphism of TRPM6 was associated with an increased risk of poststroke epilepsy. TRPM6C genotype (CC) frequency was significantly higher in those patients than in controls (*P* = 0.006). The mutant allele with the C variant is more common in patients with poststroke epilepsy. TRPM6 are essential regulators of Mg2+ transport. Mutated TRPM6 gene results in hereditary hypomagnesemia [53]. Therefore, the mechanisms of ictogenesis may be achieved by decreasing the magnesium ion concentrations, which leads to a reduced seizure threshold and an increased vulnerability to epilepsy. This phenomenon that sera magnesium content generally decreased seems to be confirmed in vivo in epilepsy patients [54]. Compared with the TT genotype, the TRPM6rs2274924 CC genotype was significantly associated with an induced risk of poststroke epilepsy. The frequency of the C allele in poststroke epilepsy patients was significantly higher than that in the nonepilepsy group [55].

### 4.3. CD40/CD40L System

Mounting evidence implicates that inflammatory response and oxidative stress are implicated in the pathogenesis of poststroke epilepsy, while agents having antioxidant activity also exert neuroprotective effects in seizures. The interaction of CD40 with its ligand, CD40 ligand (CD40L), is the link between inflammation and immunity [56]. On the one hand, activated CD40 initiates the inflammatory response via activating JNK and nuclear factor-kappa B (NF-*κ*B) pathways. On the other hand, it promotes the production of reactive oxygen species, which in turn causes the occurrence of oxidative stress [57]. Previous studies show that SCD40L is a surrogate biologic marker for the occurrence of cardiovascular risk [23, 58]. Recent retrospective studies have found that CD40 gene polymorphism is associated with seizure susceptibility. The T allele was associated with significantly increased poststroke epilepsy risk compared with the C allele. Furthermore, this CD40T gene exhibits a stronger expression of mRNA and the content of CD40 protein and the ligand in the carriers were significantly higher than those in other groups, which is particularly apparent in the TT group. CD40L is also thought to be a prognostic marker for unfavorable outcomes [59]. Elevated CD40L levels were associated with a worse prognosis in poststroke epilepsy patients. The CD40 system determination of stroke patients would provide more accurate and reliable information for the prediction of the occurrence and prognostic of poststroke epilepsy.

## 5. Neuroprotein Biomarkers

### 5.1. S100B

S100 calcium-binding protein B (S100B) is a Ca2+ binding protein that is expressed mainly in the brain by astrocytes, which participates in signal transduction, inflammatory reactions, and repair of neurons and glial cells after central nervous system injury. Previous research has highlighted the importance of S100B not only as the most useful biomarker for glial cell injury and central nervous system dysfunction but also as a tool for the diagnosis and prediction of epileptic seizures [60, 61]. Although the inclusion criteria are different, many studies have shown that the serum S100B in epilepsy patients is significantly higher than that in the control group regardless of the aetiology and location of the seizures, and this increases with increased frequency of seizures. The central nervous system damage and the inflammatory response caused by a stroke can stimulate the expression of S100B. The severity of the ischemic injury and the stimulation by microbial components that are risk factors for poststroke epilepsy has been widely acknowledged and endorsed. Therefore, it would be plausible for S100B to be increased in these patients. Before thrombolytic therapy in high-risk patients undergoing hemorrhagic transformation, elevated serum S100B levels were observed [62]. In 1070 days median follow-up for 90 patients on mechanical thrombectomy treatment, Eriksson proved that multiple serology indicators, including S100B in patients with poststroke epilepsy, are higher than normal. With a sensitivity of predicting poststroke epilepsy being 100%, the best specificity can still reach 77%~93%. However, given the low incidence of poststroke epilepsy in this study, statistical analysis was not further performed [63]. Recent meta-analyses of several cohorts provide a multitude of evidence that support the elevation of S100B protein level in epilepsy patients. Two recent meta-analyses also provided evidence for the increase of serum S100B levels in patients with epilepsy. S100B is the most valuable biological marker for seizures and prognosis [64, 65].

Some researchers have come out with the opposite conclusion. Abraira concluded that reduced S100B levels were related to a greater risk of epilepsy after stroke. S100B level < 1.364 (*P* = 0.001) is an independent predictor of poststroke epilepsy [66]. S100B plays a crucial role in maintaining the integrity of the blood-brain barrier function. The reduction of S100B not only causes the disorder of the blood-brain barrier function but also leads to the imbalance of microglia function that both will induce epilepsy [67]. Although there are many studies on S100B, the mechanism by which S100B leads to epilepsy is unclear. S100B in patients with ischemic stroke did not increase until eight hours after the ischemic damage, while the serological samples in Zhang et al.'s study were drawn within six hours after stroke [68]. Whether the levels at different times have different significance for poststroke seizures still needs to be confirmed by large-scale clinical trials.

### 5.2. Hsc70

Heat shock 70 kDa protein-8 (Hsc70) is a member of the heat shock protein 70 (Hsp70) family, which is a potential predictor of poststroke epilepsy. A retrospective study of 14 serological blood indicators in 1115 stroke patients found that heat shock protein levels in patients with early poststroke epilepsy were higher than those without seizures and the control group, although the difference did not reach statistical significance after adjusting for confounding factors [42, 63]. Another retrospective study of late poststroke epilepsy also found that the heat shock protein was significantly decreased in the epileptic group, and the low level of heat shock protein is a biomarker that can be used in the prediction and detection of poststroke epilepsy. ROC curve analysis showed that the optimal cut-off value of the seizure risk is 2.496. Hsc70 has neuroprotective effects, which act as molecular chaperones, promoting the correct folding of proteins to maintain protein homeostasis [66, 69]. Decreasing Hsc70 contributes to impairment of blood-brain barrier integrity, altering the susceptibility of poststroke epilepsy.

In addition, the decrease of Hsc70 may be related to the increased expression of Hsp70 in poststroke stress [70]. Heat shock protein 70 (Hsp70) is a stress protein and is expressed after exposure to various stress conditions, which is significantly higher in patients with temporal lobe epilepsy than in normal subjects. Some studies have also suggested that heat shock protein 70 is a stress marker of temporal lobe epilepsy. Those who had more frequent seizures also reported expressing more Hsp70 compared to those who had less frequent seizures [71]. In particular, the expression levels of Hsp70 in the hippocampus are positively correlated with epileptogenic power and negatively correlated with surgical treatment efficacy. This could be due to the hyperexcitability of cortical networks caused by the strong expression of heat shock proteins under stress. At present, reports on the study of Hsp70 in poststroke epilepsy are scarce. In particular, the roles of Hsp70 in seizure activity are still controversial.

The fact that specific alterations of different biomarkers were observed in epilepsy patients supports the notion that biomarkers can improve the prediction of patients who later experience acute or remote symptomatic seizures after stroke and could be valuable in addition to clinical or EEG factors. Besides, biomarkers may also have important reference significance for understanding the mechanism of epileptic seizures and evaluating prognosis.

## 6. Conclusions

In summary, due to progress in treatments of poststroke patients, the prevalence of stroke-related seizures is increasing. Although the EEG is strongly recommended to monitor epileptic activity, there are still many difficulties in clinical application. The current guidelines only give some weak recommendations on prevention of occurrence of poststroke seizures, and the use of antiepileptic drugs remains controversial. Hence, the use of biomarkers in the prediction of poststroke epilepsy provides a complementary approach. In this review, it is divided into inflammatory biomarkers, neurotransmitter biomarkers, genetic biomarkers, and neuroprotein biomarkers. Changes in the levels of these biomarkers are largely related to the occurrence and prognosis of PSE. The discovery and application of biomarkers will identify new pharmacological targets and develop more therapies for the treatment of PSE. Further large-scale clinical randomized controlled trials are required to support the research results.

## Data Availability

The data used to support the findings of our study are included within the article (see References).
